# Paralytic and Amnesic Shellfish Toxins Impacts on Seabirds, Analyses and Management

**DOI:** 10.3390/toxins13070454

**Published:** 2021-06-29

**Authors:** Begoña Ben-Gigirey, Lucía Soliño, Isabel Bravo, Francisco Rodríguez, María V. M. Casero

**Affiliations:** 1 Centro Oceanográfico de Vigo (IEO, CSIC), 36390 Vigo, Spain; lucia.solino@ieo.es (L.S.); isabel.bravo@ieo.es (I.B.); francisco.rodriguez@ieo.es (F.R.); 2 RIAS Wildlife Rehabilitation and Research Centre, Parque Natural da Ria Formosa, 8700-194 Olhão, Portugal; mariavmcasero@gmail.com

**Keywords:** seabirds, mass mortality events, wildlife management, paralytic shellfish toxins, PSTs, amnesic shellfish toxins, ASTs, analyses, HABs, vectors

## Abstract

Marine biotoxins have been frequently implicated in morbidity and mortality events in numerous species of birds worldwide. Nevertheless, their effects on seabirds have often been overlooked and the associated ecological impact has not been extensively studied. On top of that, the number of published studies confirming by analyses the presence of marine biotoxins from harmful algal blooms (HABs) in seabirds, although having increased in recent years, is still quite low. This review compiles information on studies evidencing the impact of HAB toxins on marine birds, with a special focus on the effects of paralytic and amnesic shellfish toxins (PSTs and ASTs). It is mainly centered on studies in which the presence of PSTs and/or ASTs in seabird samples was demonstrated through analyses. The analytical techniques commonly employed, the tissues selected and the adjustments done in protocols for processing seabird matrixes are summarized. Other topics covered include the role of different vectors in the seabird intoxications, information on clinical signs in birds affected by PSTs and ASTs, and multifactorial causes which could aggravate the syndromes. Close collaboration between seabird experts and marine biotoxins researchers is needed to identify and report the potential involvement of HABs and their toxins in the mortality events. Future studies on the PSTs and ASTs pharmacodynamics, together with the establishment of lethal doses in various seabird species, are also necessary. These studies would aid in the selection of the target organs for toxins analyses and in the postmortem intoxication diagnoses.

## 1. Introduction

Phytoplankton is fundamental to the functioning of marine ecosystems. Primary producers fuel the food chain from microzooplankton to invertebrates, fish, aquatic seabirds and mammals. Though, under favorable conditions, uncontrolled growth can lead to harmful algal blooms (HABs) with toxic/deleterious effects to wildlife and humans [1,2,3] through the consumption of contaminated sea products (e.g., shellfish and fish).

HAB episodes are apparently increasing both in frequency and intensity, expanding their geographical distribution over the last decades [4]. Aquaculture intensification in coastal waters, eutrophication processes, transport of dinoflagellate cysts in ballast water or during the translocation of shellfish stocks and climate change have been cited behind those trends [1,5,6,7,8,9,10]. However, a recent meta-analysis of HABs [11] did not evidence a global increase in the last three decades, but rather intensified monitoring combined with emerging HAB syndromes or impacts.

Research and monitoring of HABs have benefitted in recent decades from technological advances for remote and in situ detection, as well as from the improvement of analytical methods for marine biotoxins (e.g., [6,12,13,14,15,16]). 

The extent and degree to which HABs negatively affect marine organisms, such as seabirds, are related to the fate of algal derived secondary metabolites (toxins or bioactive compounds) in the ecosystem and the biological activity and bioavailability of those substances [17]. Some harmful algae do not produce toxic secondary metabolites, but can still cause direct or indirect mortalities by physical mechanisms [17,18]. Other species produce potent toxins during blooming, which can be accumulated by filter feeders [19]. Within marine ecosystems, harmful algal toxins can be transmitted through the food web from zooplankton to different fish, marine invertebrates (gastropods, crustaceans, equinoderms, tunicates), seabirds, marine mammals, and people [20,21,22,23]. Marine biotoxins pose a serious threat to human health. The ingestion of contaminated seafood can produce syndromes with varying degrees of severity such as paralytic shellfish poisoning (PSP) and amnesic shellfish poisoning (ASP), among others [24,25,26,27]. As a rule, it is the digestive tract of vector species (shellfish, crabs, snails, fish, etc.) that contains the highest biotoxin concentrations, not the muscle tissue. Therefore, the risk of intoxication is highest from seafood eaten without removing the digestive tract, such as bivalve mollusks or whole fish eaten by seabirds and marine mammals.

Marine biotoxins have been frequently implicated in morbidity and mortality events in numerous species of birds worldwide [10,28,29,30,31,32]. Nevertheless, their impacts on seabirds have not been extensively studied, particularly in the context of spatial and temporal links between seabird mortality events and biotoxins. 

Marine biotoxin effects on seabirds, common and important members of aquatic ecosystems, have often been overlooked or only casually mentioned [29,33]. This is partially due to the difficulties to establish a direct link with HABs, because wrecks may be detected once the causative organism has already faded out. Another reason could be the fact that seabirds may ingest prey that accumulated marine biotoxins during HAB events that already vanished. In this sense, it is also important to highlight the low number of published studies confirming, by analyses, the presence of marine biotoxins from HABs in seabirds.

Nowadays, the advances in analytical methods with increased sensitivity and selectivity [12,13,14,16,25], allow seabird carcasses to be tested for the presence of marine biotoxins. To this end, relevant tissue samples need to be taken and sampling preparation must be adapted, if required, to the particular tissues [34,35,36,37,38]. However, the ultimate cause of seabird’s morbidity and mortality cannot always be found. To tackle this issue, joint efforts of researchers studying toxic phytoplankton and marine biotoxins, together with veterinarians, ornithologists and staff from wildlife hospitals (and other organizations for the study and conservation of avifauna), should be encouraged.

The goal of this review is to compile information on studies evidencing the impact of HABs on marine birds, paying special attention to the effects of paralytic shellfish toxins (PSTs) and amnesic shellfish toxins (ASTs). We aim to focus mainly on those studies in which the analyses of marine biotoxins in seabirds allowed for to suggest a cause–effect relationship. Another goal is to summarize the analytical techniques employed and the possible adjustments in protocols for processing seabird samples. The key role of the different vectors in the intoxication of seabirds is also discussed. Moreover, we intend to collate the information available on clinical signs and pathology in birds affected by PSTs and ASTs, mentioning as well other causes that can aggravate the syndromes. All these data can be very helpful for staff involved in the rescue and treatment of the affected species. Finally, the importance of having adequate management plans in the case of mortality events and the need to foster collaborations between the different organizations involved in the protection of seabirds and other researchers (i.e., marine biotoxin experts) is highlighted.

## 2. Direct and Indirect Impacts from HABs on Marine Birds. Biotoxins and Other Bioactive Compounds

Most marine HABs are associated with a few groups (e.g., dinoflagellates, diatoms, raphidophytes, pelagophytes and haptophytes) that produce secondary metabolites potentially deleterious to other organisms [17]. The broad spatial coverage, trophic transfer, and temporal persistence of HAB toxins create a wide range of direct and indirect lethal/sublethal effects on marine life in general, and on seabirds in particular. However, apart from biotoxins, there exist other bioactive algal compounds harmful to seabirds that do not bioaccumulate, or biomagnify, in the food chain. For example, the production of an oily substance by a bloom of the diatom *Coscinodiscus concinnus* in spring 1996 in the southern German Bight resulted in stranding of red-throated divers (*Gavia stellata*), due to plumage contamination [28]. Moreover, in summer–autumn 2009, the death of thousands of seabirds (e.g., surf scoters (*Melanitta perspicillata*), common murres (*Uria aalge*), pacific loons (*Gavia pacifica*), and western grebes (*Aechmophorus occidentalis*) off Washington and Oregon states was attributed to a proteinaceous foam after the decline of a bloom of the dinoflagellate *Akashiwo sanguinea* [39,40,41]. In this regard, Jessup et al. [42] reported another *A. sanguinea* bloom causing unprecedented beach strandings of live and dead seabirds in California, with 14 species recorded. The foam after these blooms contained surfactant-like proteins that destroy the waterproof and insulating layers of feathers. As a result, restricted flight, hypothermia, starvation and stress can happen in birds, which eventually die [41].

Marine and freshwater toxins derived from HABs have been associated with morbidity and mortality events for numerous species of birds in various parts of the world [29,30,31,32,43,44,45,46,47,48,49]. Most episodes have been reported in North America (Canada, USA) and Europe. Important seabird mortalities have also been recorded in other continents/countries, although these events might be underreported. For instance, Stephen and Hockey [50] revealed that at Penguin Island (Lamberts Bay, South Africa), HABs were the fourth most important cause of seabird mortality and the primary cause of mortality for gulls (*Larus* spp.) and terns (*Sterna* spp.) from 1997–2002. Marine biotoxins may also indirectly affect seabirds by poisoning prey resources, incurring starvation or relocation for resident seabirds [32]. Such effects may be particularly pronounced on nearshore species feeding on benthic organisms that accumulate marine biotoxins. While some shorebirds may be able to discriminate between prey with different concentrations of toxins [51,52], large blooms could hamper efficient relocation, and changes to foraging range and efficiency may affect reproduction [53]. The three main groups of marine biotoxins involved in seabird morbidity and mortality worldwide are brevetoxins (PbTXs), PSTs and ASTs. We will briefly describe the impact of these toxins on seabirds. However, given the relevance of ASTs and PSTs in Europe, we will mainly focus this review on them.

### 2.1. PbTXs 

HABs of the brevetoxin-producing dinoflagellate *Karenia brevis*, also known as “Florida red tide”, are periodically reported in the Mexican Gulf and coastal waters of Ecuador [17,34,49,54], where seabird mass mortality events (MMEs) have been associated with PbTXs. On admission for rehabilitation, birds had neurological clinical signs, including loss of palpebral reflex, loss of anal tone, inability to stand, inability to lift head, disorientation, head tilt, head tremors, ataxia, and seizures [34]. From 2005–2007, Van Deventer [30] conducted an important study to evaluate the accumulation of these toxins in the tissues of seabirds and their prey items. Their results indicated that piscivorous marine birds, including double-crested cormorants (*Phalacrocorax auritus*), brown pelicans (*Pelecanus occidentalus*), terns and gulls were exposed to a range of PbTxs levels in their diet during *K. brevis* blooms. Direct ingestion appeared to be the primary route of exposure, as PbTxs-contaminated fish were confirmed in the stomachs of several birds. Shorebirds and gulls could have also been exposed to PBTxs via the scavenging of red tide-killed fish deposited on beaches during blooms.

### 2.2. PSTs

PSTs are mostly associated with marine dinoflagellates (genera *Alexandrium*, *Gymnodinium* and *Pyrodinium*) and freshwater cyanobacteria, which form extensive blooms around the world [25]. Binding of PSTs to voltage-gated sodium channels and the blockade of ion conductance through these channels is the major molecular mechanism of action of this group of toxins on nerves and muscle fibers [55]. As a consequence, a progressive loss of neuromuscular function ensues, leading to the reported neurotoxic symptoms that could eventually result in death by asphyxia. The syndrome is known as PSP. Seabird MMEs involving PSTs originating from *Alexandrium* spp. have been documented in North America and Europe, usually where piscivorous birds consumed contaminated fish [29,37,44,45,46,56,57,58,59,60]. In a review by Band-Schmidt et al. [61] about the taxonomy, bloom dynamics, toxicity, autoecology, and trophic interactions of PSTs producing dinoflagellates in Latin America—some episodes in which seabirds were affected are mentioned. Potentially, any species is susceptible to this harm if exposed to high concentrations through the food chain [17]. As with other fauna, concerns for threatened seabird species are particularly high. For instance, Stephen and Hockey [50] attributed the mortality of 53% of the local African black oystercatcher (*Haematopus moquini*) population and gulls to a toxic bloom of *A. catenella* in Saldanha Bay, South Africa, in 1978.

Earlier reports of seabirds mass mortalities attributed to PSTs describe red tide events that also triggered outbreaks of illness in humans and many different organisms in the US states of Washington and Massachusetts and on the UK northeast coast (see [29] for detailed historical records). Several bird species were affected in the different episodes, such as common shags (*Phalacrocorax aristotelis*), black ducks (*Anas rubripes*), terns, black-footed albatross (*Diomedea nigripes*), pacific loons, northern fulmars (*Fulmarus glacialis*), tufted puffins (*Fratercula* *cirrhata*), various gull species, etc. In some of these events, authors could not establish true cause and effect [29,56]. In other episodes, PSTs toxicity was quantified only in bivalve mollusks and/or fish [44,45,46,57]. Nevertheless, the occurrence of *Alexandrium* HABs and the symptoms observed in seabirds pointed to PSTs as the causative agent. PSTs were likely ingested via prey vectors (shellfish, crustaceans and fish). The fact that Coulson et al. [44] had been studying seabirds in the affected UK region for several years favored the provision of one of the few in-depth reports on the impacts of HABs on bird populations [29]. It was estimated that around 80% of the breeding shags population died in Northumberland [44,57]. In 2011–2012, up to 21% of Kittlitz’s murrelet (*Brachyramphus brevirostris*) nestlings died shortly after consuming sand lance (*Ammodytes hexapterus*), a fish species known to biomagnify saxitoxin [60]. Upper gastrointestinal content, liver, and kidney samples from chicks were analyzed for STX. The toxin was detected in 7 out of the 8 samples tested (see Table 1). An important study that provided strong evidence for the trophic transfer of PST resulting in mortalities of multiple wildlife species was conducted by Starr et al. [35], after an intense *Alexandrium* bloom in St. Lawrence Estuary (Canada) in August 2008. This bloom caused the death of many seabird species. Pathological analyses were performed on a total of 74 birds of 13 species and lesions were consistent with PSP respiratory paralysis. Significant PST levels (Table 1) were found in the liver and/or the gastrointestinal contents of several seabird carcasses tested, as well as in live planktivorous fish, mollusks and plankton samples collected during the bloom. The authors suggest that such mortalities are expected to increase in the future as the frequency, intensity and geographic extent of toxic algal blooms are increasing worldwide. More recent studies on MMEs caused by PSTs are mentioned in Section 6, with details on species affected, tissues selected and PSTs levels shown in Table 1.

### 2.3. ASTs

ASTs (DA and its isomers) are a group of marine biotoxins of which DA is the main compound. ASTs are produced only by diatoms (mainly the genus *Pseudo-nitzschia*, but also some *Nitzschia* and *Amphora* species) and certain rhodophytes [65,66]. DA can bioaccumulate in the tissues of marine organisms, such as shellfish, anchovies and sardines that feed on the phytoplankton able to produce this toxin. Thus, other marine animals, seabirds, or even humans could exhibit an acute intoxication via the consumption of contaminated foods [19]. This syndrome is known as ASP and causes effects on both the gastrointestinal tract and nervous system.

Shellfish toxicity due to domoic acid (DA) was discovered in 1987 in Canada, when three people died and 105 became ill from eating contaminated blue mussels [67]. The first documented ASP outbreak happening in 1991 in Monterey Bay, California (CA) caused the death of dozens of brown pelicans and Brandt’s cormorants (*P. penicillatus*) [47]. DA was detected in the stomach contents of dead and sick pelicans and cormorants, as well as in anchovies that may have acted as vectors of DA produced by *Pseudo-nitzschia australis*. Sierra-Beltrán et al. [48] reported a mortality episode of approximately 150 brown pelicans during the winter of 1996 in Baja California Peninsula (Mexico). Deaths were associated with the consumption of mackerel (*Scomber japonicus*) contaminated with DA. Other cases of DA toxicity in birds have been documented by several authors [18,29,68]. Since 2003, hundreds of bird strandings or deaths from central to southern CA have been attributed to DA and there is evidence that these poisonings are increasing [31]. 

Nevertheless, the first birds’ massive stranding associated with DA in that region could have been the one revisited by Bargu et al. [69]. In Santa Cruz (CA) in 1961, a local newspaper reported thousands of seabirds (sooty shearwaters, *Puffinus griseus*) on the shores of North Monterey Bay. The animals were seen regurgitating anchovies, flying into objects and dying on the streets. Alfred Hitchcock, a summer resident in the area, contacted a local newspaper requesting a copy of their article published on August 18th. “The birds” was released two years later, based on Daphne du Maurier’s novel, using the report of the 1961 event as research material for the film. Bargu et al. [69] examined the archival samples of herbivorous zooplankton at the time of the bird frenzy and found the dominance of DA producing diatoms (several *Pseudo-nitzschia* species). The authors estimated that these diatoms attained similar numbers to those during recent stranding events due to DA poisoning in the area, being likely responsible for the 1961 episode by the accumulation of that toxin in the food chain. Table 2 compiles information on MMEs linked to ASTs, including data on species affected, tissues selected, and AST concentrations quantified.

## 3. Vectors Involved in ASTs and PSTs Toxins Transmission to Seabirds

**Figure 1:**
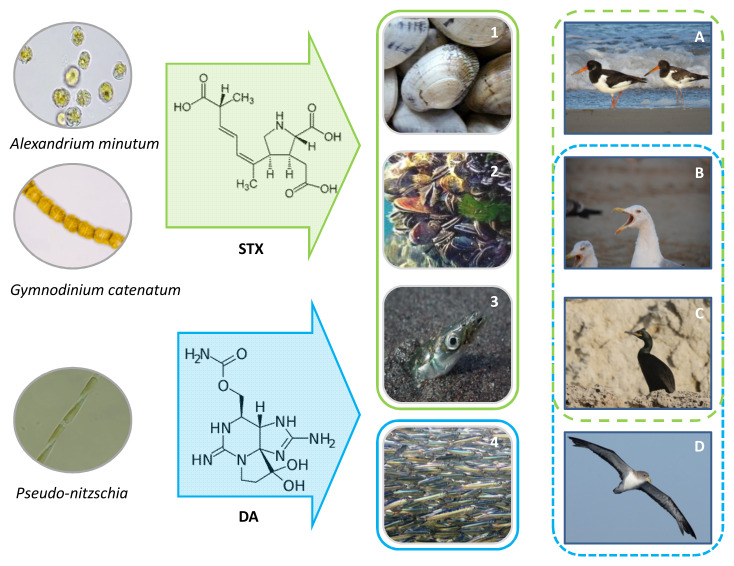
Conceptual diagram of most common vectors of marine biotoxins associated with seabird poisonings. Green lines represent the most common PSTs vectors (bivalves and certain fish) and potential seabird groups affected by them (oystercatchers, gulls and shags, among others). Blue lines represent the most common DA vectors (pelagic fish) and potential seabird groups impacted (mainly pelagic seabirds). Pictures of vectors: 1. *Venerupis pullastra,* 2. *Mytilus galloprovincialis,* 3. *Ammodytes hexapterus* (extracted from: Wikimedia Commons, author: Mandy Lindeberg, NOAA/NMFS/AKFSC), 4. *Engraulis encrasicolus* (extracted from: fishbase, author: Alessandro Duci). Seabird pictures: (**A**) *Haematopus ostralegus*, (**B**) *Larus michahellis*, (**C**) *Phalacrocorax aristotelis*, (**D**) *Calonectris borealis* (author: Pere Josa).

Marine biotoxins are usually transferred in the marine trophic chain after the ingestion of toxin-contaminated primary and secondary consumers (filter and suspension feeders, including epipelagic and benthic fauna) [70]. These organisms, exposed directly to toxic microalgae, are the vectors that concentrate, biotransform and/or biomagnify the toxins in the food web, which end up ingested by top predators like marine mammals, seabirds and humans (Figure 1).

From herbivorous microzooplankton and metazoans (copepods, shrimps, jellyfish, fish larvae, etc.), to a variety of suspension and filter-feeding shellfish, cephalopods, crabs, echinoderms, snails, bryozoans, etc., as well as planktivorous fish (herring, mackerel, sardine, anchovies, etc.), they can all consume and retain toxic microalgae, incorporating and potentially transferring their toxins in the food chain [22,23,71,72,73,74,75,76,77]. With regard to ASTs and PSTs, as well as for other biotoxins, some of these vectors convert the original compounds from the microalgae to other congeners, to eliminate them and detoxify their tissues [18,35,78,79].

Table 3 lists the dinoflagellates and vectors involved in PSP outbreaks linked with seabird mortalities. Such episodes have only been reported in the Pacific and Atlantic coasts of North America and UK. All of them were associated with *Alexandrium*, but until its formal redescription by Balech [80], previous studies referred to some species, as in the genus *Gonyaulax*. In South America, the mortality of seabirds (penguins, seagulls, terns, cormorants, ducks, grebes) has been observed along the Buenos Aires, Patagonian, and Beagle Channel coasts, often related to *A. tamarense/catenella* blooms [81]. Furthermore, the *A. catenella* bloom in 2016 in the Pacific Chilean coast triggered massive mortalities in invertebrates, mammals and birds [43,82] (and references therein). Recently, Pitcher et al. [83] summarized that PSTs have also been related with seabird mortalities in South Africa [84]. These authors also mentioned observations back to 1901 about hundreds of dead cormorants (*Phalacrocorax* spp.) floating together with tons of dead sardines in “muddy” colored water in St. Helena Bay, likely due to the transfer of PSTs from a bloom of *A. catenella* [85]. Finally, Pitcher et al. [83] referred to bird mortalities with symptoms of PSP on remote islands off Namibia, although the exact causes have never been confirmed.

Among the diatoms able to produce DA, only *Pseudo-nitzschia* has been reported to cause severe impacts on aquatic ecosystems, including diverse marine fauna and seabirds [86,87]. According to the available literature, ASP intoxications in seabirds (Table 4) have been restricted to the Pacific coast of North America (California and Mexico), always associated with distinct assemblages of *Pseudo-nitzschia* spp.

**Table 3:** Potential vectors and phytoplankton species involved in seabird mortality events associated with PSP outbreaks.

Vectors	Affected Birds, Place and Dates	Phytoplankton Species	Observations	References
Clams, barnacles and other benthic mollusks	Common murres, pacific loons, gulls, white-winged scoters and others (Washington coast, USA); May 1942	*Gonyaulax catenella*	Coincidence with PSP outbreak	[56]
Shellfish (e.g., mussels, clams)	Mostly shags but also: cormorants, terns, fulmars and others (Farne Islands, Northeastern England); May 1968 and spring 1975	*Gonyaulax tamarensis*	Toxicity not determined in birds, only in shellfish samples collected	[44,45,57,88]
Filter-feeding bivalves (e.g., mussels and clams)	Black ducks, waterfowls, gulls and other shorebirds (from southern Maine to Cape Ann, USA); September 1972	*Gonyaulax*	Toxicity not determined in birds, only in shellfish samples collected	[89,90,91]
Sand lances	Common terns, arctic terns, roseate terns, laughing gulls, herring gulls (Cape Cod, USA); June 1978	*Gonyaulax*	PSTs only determined in sand lance	[46]
Mussels	Black oystercatchers, southern blackbacked gulls, Hartlaub’s gulls (South African coast); May 1979	*Gonyaulax catenella*	Birds with internal lesions and empty stomachs, probably starved to death	[50,92,93]
Sand lances	Herring gulls (St. Lawrence Estuary, Canada); July 1996	*Alexandrium*	PSTs in sand lance and in bird intestine and brain	[58]
Mollusks and planktivorous fish (e.g., sand lance and capelin)	15 species, mostly larids especially Black-legged kittiwakes (St Lawrence Estuary, Canada); August 2008	*Alexandrium tamarense*	PSTs in bird carcasses, mollusks, planktivorous fish, and plankton	[35]
Sand lance (birds died after eating them)	Nestlings of kittlitz murrelets (Alaska, USA); 2011 and 2012	*Alexandrium*	STX detected in sand lances and 87% of nestling carcasses	[60]
Euphausiids and forage fish (e.g., sand lance, capelin, herring, juvenile pollock)	Common murres (Alaska, USA); 2015 and 2016	*Alexandrium catenella*	PSTs detected in fish, invertebrates and in birds in which could be a secondary cause of death	[37]
Unknown	Northern fulmars, short-tailed shearwaters and murres, among others (Bering Sea and Chukchi Sea, Alaska, USA); June–September 2017	Unknown	PSTs detected in carcasses. PSTs along with starvation probably caused bird die-off	[62]
Not reported	Common murres, surf scoters, white-winged scoters, Brandt’s cormorants, brown pelicans, double-crested cormorants, northern fulmars; several Washington and California counties, USA; September–October 2009, July 2015–March 2016, 2018	*Alexandrium sp.* present in some areas	Low PSTs levels detected in carcasses	[64]

## 4. Symptoms of PSP and ASP Intoxications in Seabirds/Birds

Although the reports of sick or dead birds during HABs are increasing, only a few studies have compiled the information on the acute or differential effects of marine biotoxins on bird communities and populations. Seabirds exhibit a wide range of sensitivities to algal toxins and symptoms vary depending on each species and the microalgae involved [29]. An excellent review on the symptoms is that of Landsberg et al. [59]. These authors summarized the information on the most common HABs marine biotoxins, providing descriptions on clinical signs, pathology and circumstances, as reported in wild bird mortalities. Symptoms reported in the literature for PSP and ASP in seabirds are summarized in Table 5 and Table 6, respectively.

An important point to consider is the possibility of confusing the PSTs effects with those of other neurotoxic compounds, such as pesticides, or botulism [45]. Natural toxins, infectious diseases and industrial chemicals have been associated with neurotoxicity in birds [96]. A table in their article compiles all the factors that could cause avian paralysis, ranging from biotoxins (botulism and PSTs), to nutritional deficiencies, to environmental contaminants (heavy metals, organochlorines, organophosphates) and to infectious diseases.

**Table 5:** Symptoms and pathological lesions of PSP in dying seabirds.

Symptoms and Lesions	Symptoms and Lesions Details	Affected Birds	References
**Neurological symptoms**	Loss of equilibrium (inability to stand or even keep head up)	common murres, shags, terns, gulls, cormorants, eiders	[35,44,46]
	Uncoordinated movements (ataxia)		
	Falling forward		
	Unable to take off		
	Convulsions		
	Mild to severe paralysis		
	Unable to move wings or legs		
	Paralysis in the oviduct		
**Eye symptoms**	Pupil restriction	Shags	[44]
**Gastrointestinal symptoms and lesions**	Excess vomiting, food regurgitation	Gulls, white-winged scoters, shags, terns	[35,44,46,56]
	Abnormal feces (i.e.: greenish, yellowish, brownish)		
	Excessive defecation		
	Protruding cloaca		
	Inflamed alimentary canal. Congestion of tracheal and oral mucosa		
	Intestinal inflammation and/or hemorrhage		
	Thickened duodenal or intestinal mucosa and pale mucoidal material		
**Circulatory and respiratory problems**	Distended or dilated veins	Shags, terns	[35,44,46,57]
	Hemorrhages at the base of the brain or elsewhere in the body		
	Failure of circulatory system.		
	Congestion of organs, including lungs		
	Frequent gasping		
**Starvation**	Weight loss	Shags	[44]
	Loss of subcutaneous fat		
**Other**	Inability to lay eggs	Terns	[44]

**Table 6:** Symptoms and pathological lesions of ASP in dying seabirds.

Symptoms and Lesions	Symptoms and Lesions Details	Affected Birds	References
**Neurological symptoms and lesions**	Slow side-to-side head waving	Brown pelicans, Brandt’s cormorants, common murres, sooty shearwaters	[33,47,48,69,97]
	Ventroflexed head		
	Torticollis		
	Wings partially extended		
	Motor tremors		
	Unable to take off		
	Inability to retract legs during flying		
	Clenching of toes		
	Scratching		
	Disorientation and loosing awareness of their surrounding		
	Loss of equilibrium (inability to stand or keep head up)		
	Uncoordinated movements (ataxia)		
	Falling on their back or side with feet paddling		
	Abnormal behavior (agitation or unusually docile, asocial behavior and irresponsiveness to handling)		
	Diffuse neural necrosis		
	Capillary endothelial cell hyperplasia		
	Myofiber necrosis in the right ventricular wall		
**Gastrointestinal symptoms**	Vomiting, food regurgitation	Brown pelicans, Brandt’s cormorants, sooty shearwaters	[47,69]
**Circulatory and respiratory problems**	Focal hemorrhages at the adductor, sartorius, gracilis and vastus medialis muscles of the hind limb and the biceps brachii of the forelimb	brown pelicans, Brandt’s cormorants,	[47]
	Vascular engorgement of the intestine		
**Starvation**	Weight loss	Common murres	[98]
	Loss of subcutaneous fat		
**Paralysis**	Decreased mobility and responsiveness to stimulus	Common murres	[97]
	Weakness and lethargy		
**Other**	Focal muscle necrosis	Brown pelicans, ommon murres	[47,97]
	Elevated serum creatinine kinasa, blood urea nitrogen and uric acid		
	Hypothermia		
	Necrosis of pectoral muscles		
	Dark-brown urates		

## 5. Multifactorial Causes of Seabird’s MMEs

The term wreck is commonly used to name frequent large strandings of dead or moribund birds. Wrecks washed up on beaches can be explained by adverse weather, food shortage, pollution (i.e., chemical pollution), fishing activities (i.e., bycatch, entanglement in nets and fish traps), mariculture (drowning of seabirds in fish pens) and parasites [28,99]. While the main causes of seabird deaths are storms, oil, severe cold weather and lack of food, and other reasons can include various chemical pollutants, toxins, calm weather, diseases and parasites [96,100]. A study [99] reported that some causes of mortality (i.e., oil, weather, chemical pollution, etc.) often act in synergy. It is also important to note that MMEs are increasing in frequency and magnitude, potentially linked with ongoing climate change (persistent warming) [63,101].

On many occasions, seabird wrecks cannot be associated with a single cause, since the accumulating effect of various factors may trigger mass mortalities. For instance, adverse weather conditions may affect foraging behavior and success and may be indirectly responsible for wrecks of emaciated specimens [28]. Some literature examples include marine biotoxins as potential cofactors. Low PST or AST levels induce a loss of motor coordination in seabirds leading to impaired swimming, flying, foraging and death by starvation [29,44]. A research article [10] evaluated the extreme mortality and reproductive failure of common murres resulting from the northeast Pacific marine heat wave of 2014–2016. Increased ocean temperatures during and following such events were associated with HAB development [102]. In particular, an extensive bloom of *Pseudo-nitzschia* spp., including DA producers, was documented in coastal California from March through June 2015 [75]. This event led to the bioaccumulation of DA in northern anchovies (*Engraulis mordax*), one of the main preys of common murres. Low DA levels were found in tissues of beach cast common murres during and after the 2015 bloom. Nonetheless, Gibble et al. [98] concluded that starvation was likely the ultimate cause of death and that the oceanographic conditions together with HAB effects were secondary. Low STX levels were detected in stomach or cloacal contents of all four tufted puffins analyzed in an anomalous mortality event [63]. Collected specimens were severely emaciated, suggesting starvation as the ultimate cause of death. However, although acute toxicosis was not diagnosed in these birds, it cannot be entirely ruled out, due to the small number of birds tested. 

It is still unclear whether PSTs played any role in the high mortality rates of common murres during the Alaska 2015–2016 heatwave. Deaths were mainly attributed to starvation [10]. Trace STX levels were detected in 20% of 39 samples tested at the National Oceanographic and Atmospheric Administration (NOAA). Further analyses of 56 murres (including die-off and healthy specimens), forage fish and invertebrate prey collected in 2015–2017 were conducted at the U.S. Geological Survey (USGS), Alaska Science Center. Results indicated a low to moderate frequency (20–54%) of STX occurrence among taxa groups, but all at relatively low concentrations [37]. Authors could not corroborate the hypothesis that biotoxins were the primary cause of murres mortality in Alaska, but their contribution to the die-off was not dismissed. A similar situation was reported from 2017 by Van Hemert et al. [62] in the Bering and Chukchi Seas. A total of 26 carcasses were sampled during seabird MMEs and PSTs were detected in 60% of the samples. Toxin levels in northern fulmars (*Fulmarus glacialis*) were within the range of those reported from other PST-induced bird deaths, suggesting that these toxins may have contributed to mortalities (Table 1). However, direct neurotoxic action by PSTs was not confirmed and starvation was likely the cause of death among the examined birds. Another example of potential synergistic effect is the dual exposure of seabirds to both ASTs and PSTs toxins, as reported by Gibble et al. [64]. The authors indicate that concomitant HABs may be an emerging concern, and therefore, analyses of both ASTs and PSTs are desirable in the case of MMEs potentially linked to HABs.

As indicated in Section 2, Stephen and Hockey [50] revealed that, at Penguin Island (Lamberts Bay, South Africa), HABs were the primary cause of the mortality of gulls (*Larus* spp.) and common terns (*S. hirundo*) between 1997 and 2002. The majority of tern deaths on Penguin Island occurred soon after their arrival following southward migration. The authors made the point that newly arriving migrant birds were considered particularly susceptible to HAB toxins, having depleted energy reserves and hence reduced tolerance to toxins. Stephen and Hockey [50] do not explain the reason for the lower toxins tolerance, but it could possibly be due to the higher toxins concentration per body mass when ingesting toxic prey or for the birds that are already quite weak. In their studies about the massive death of shags, Armstrong et al. [45] mentioned that elevated organochlorine pesticide residue levels reported in shags, together with their high sensitivity to PSTs, suggested the possibility of synergistic effects between both contaminants.

A study to evaluate the factors behind paralysis in wild birds was conducted by Sonne et al. [96]. They reported that it is likely that several factors (including ecological ones) play an important role, explaining why the paralysis may vary seasonally, temporally (over time), spatially (between regions) and between species. Authors also indicated that when investigating paralysis in wild birds, a holistic study approach including multiple factors is needed in order to pinpoint cause-effect relationships, as well as the potential impacts on wild bird populations. Multiple investigations have to be carried out, both in the field and in the laboratory, in order to uncover the primary cause(s). This procedure is being followed by our group that is investigating the death of around 6500 seabirds, mainly gulls, in Algarve, South Portugal from 2010–2020 [38,103]. PSTs, ASTs and botulinum toxins have already been analyzed in dead gulls, pointing to *Clostridium botulinum* as the possible cause of wrecks. Botulism may hit coastal seabirds that utilize freshwater bodies for drinking or bathing [28]. Our studies continue now with the evaluation of cyanotoxins and tetrodotoxins in additional samples. 

## 6. Determination of PSTs and ASTs Toxins in Seabirds

### 6.1. PSTs

STX and its analogs are a group of natural neurotoxic alkaloids, commonly known as PSTs [104]. The PSTs can be broadly characterized as hydrophilic or hydrophobic, and can be divided into subgroups based on substituent side chains that impart a varying level of toxicity [79]. The most representative analogue of this group is STX and the relative potency of their congeners is usually expressed by taking STX toxicity as a reference, by using toxicity equivalency factors (TEFs). The highly potent and unpredictable nature of PSTs necessitates constant monitoring of the toxin content of shellfish in the affected areas. PST monitoring programs rely on relatively intensive sampling and analysis protocols that require the availability of rapid, sensitive, accurate and precise analytical techniques [13]. We describe two main groups of methods for PSTs analysis, the ones that evaluate the sample total PSP toxicity and those that detect and quantify individual toxins.

#### 6.1.1. Methods That Evaluate Total (or Partial) Sample PSP Toxicity

##### Mouse Bioassay (MBA)

The MBA [105] is a method internationally recognized for quantifying total PSP toxicity and was the reference method for many years. The MBA detects lethal toxicity in a sample, regardless of the toxin chemical structure. Important drawbacks in the method application together with ethical considerations placed a high pressure on regulatory bodies and researchers to provide alternative methods, and it is no longer a reference method in the EU.

By 1978, Armstrong et al. (1978) [45], reported that the bioassay used to measure PSTs levels in mussels had failed to identify PSTs in shags, presumably because the concentrations in birds and fish, although lethal, did not reach the bioassay limits of detection (LOD). With regard to the STX toxicity in birds, Mons et al. [106] reported that the oral median lethal dose (LD_50_) for pigeons ranged between 91–100 µg STX equivalents·kg^−1^ body weight, which is lower than the MBA LOD. In another study, Nisbet [46] evaluated the relationship between *Alexandrium* spp. blooms and birds mortality at Monomoy National Wildlife Refuge, Massachusetts. He reported that the determination of PSTs by MBA in fish vomited by dead common terns revealed toxicity levels of 970 µg STX equivalents·kg^−1^, probably well above the lethal dose for this species. However, PSTs were not detected (LOD 400 µg STX equivalents·kg^−1^) during the analysis of two pooled liver samples from dead terns. These results suggest that low PSTs levels in terns were not detected by MBA. This is an important drawback for the use of this method in seabird samples.

##### Enzyme-Labeled Immunosorbent Assay (ELISA)

ELISA is a powerful analytical tool for natural toxins detection. Specific antibodies recognize toxins and this bound complex is quantified by labeling the free component with a reporter enzyme, usually horseradish peroxidase acting as an amplifier to produce many signals [25]. ELISA tests, such as the competitive ELISA Ridascreen Fast PSP, the Abraxis STX (PSP) ELISA or the SeaTox PSP ELISA, were developed for the quantitative determination of certain PSTs. These tests can be used for screening in a variety of shellfish, since they are fast, simple, cost effective and, for certain toxins, more sensitive than other methods. However, they may have drawbacks, such as false positive or negative results and/or the fact that the antibodies employed are selective for binding of only certain PSTs [107], and therefore do not allow the detection of all the analogs. Some of these tests have been tried in seabird samples since they are an efficient tool for screening large numbers of samples. However, further analyses of positive samples by a method that allows the detection and quantification of individual PSTs (Section 6.1.2) is desirable.

In a study conducted on Kittlitz’s Murrelets chicks samples [60], the potential presence of STX was evaluated using an ELISA assay in the Wildlife Algal-Toxin Research and Response Network (NOAA Northwest Fisheries Science Center). Matrix testing was done for each type of sample material, and minimum dilution levels were set for each sample type, in order to avoid false positive (or negative) results. A study by Jones et al. [63] described a MME affecting mainly tufted puffins, but also crested auklets (*Aethia cristatella*). A small number of puffin samples were sent to the West Wildlife Algal-toxins Research and Response Network to determine STX using an ELISA kit (Abraxis, Inc.). Although starvation was suggested as the ultimate cause of the bird’s mortality, trace STX levels were detected in all the tested samples (Table 1).

Another example of the use of ELISA for the determination of PSTs in seabird samples can be found in [35], where the ELISA for STX kit (Abraxis LLC, Warminster, PA, USA) was employed. Samples, standards and controls were processed according to the manufacturer’s instructions. However, the extraction protocol was modified to facilitate the further testing of selected samples via high performance liquid chromatography (HPLC), and to prevent the hydrolytic interconversion of toxin congeners (see Section 6.1.3), by performing all extractions in 0.1 M acetic acid. Concentrations were calculated against the standard curve response, such as described in the ELISA kit instructions. Eleven out of the 16 seabird species evaluated, showed positive results by ELISA, with STX detected in more than 67% of their carcasses. 

ELISA was also employed to test common murres samples (stomach or cloacal content) obtained in Alaska in the 2015–2016 MMEs by the National Wildlife Health Center and immediately tested at the NOAA Northwest Fisheries Science Center, Seattle, Washington, U.S. [10]. Trace STX levels were detected in eight out of 39 murre samples (Table 1). Further studies conducted by researchers at NOAA and the USGS Alaska Science Center investigated STX in a suite of tissues obtained from beach-cast murre carcasses, as well as from apparently healthy murres and black-legged kittiwakes (*Rissa tridactyla*) sampled in the previous and following summers [37]. Samples were tested by ELISA using the Abraxis STX microtiter plate assay with minor modifications. Most of the analytical methods and protocols employed for the analysis of PSTs were validated with mollusk bivalve samples. Therefore, it is very important to highlight that this study provides information on the method validation that they conducted to apply the ELISA test to seabird tissues (including the evaluation of matrix effects and recoveries). STX was detected in multiple murres and kittiwake tissues (Table 1). STX concentrations were generally lower than those reported from other studies which established a clear link between STX ingestion and bird mortality. Another study [62] also reports the use of ELISA to evaluate PSTs in samples from 26 seabird carcasses (mainly Northern Fulmars) with PSTs detected in 60% of the samples (Table 1). A recent paper [64] compiled results of STX determination conducted in seabirds in USA between 2007 and 2018 (Table 1). The studies reported were performed with either the Max Signal Saxitoxin (PSP) ELISA test kit (BIOO Scientific, Austin, Texas) or the Abraxis saxitoxin ELISA for STX kit, as per the manufacturers’ protocols. 

#### 6.1.2. Methods That Allow the Detection and Quantification of Individual PSTs

Among the main advantages of HPLC and LC-MS/MS are the higher sensitivity and selectivity and the possibility to shed light on the toxin profile of microalgae producing the bloom or samples from shellfish, fish and other PSTs vectors. Liquid chromatography (LC) is a powerful instrumental technique for the analysis of PSTs. These toxins require an oxidation in alkaline solution to produce fluorescent pyrimidine purins [108] that can be detected. A detailed review on the fluorimetric methods for the determination of PSTs that were the basis of the first LC and HPLC methods for PSTs was described by [109]. Nowadays, there are two main HPLC methods with fluorescence detection (FLD) worldwide employed for PSTs detection and quantification: an HPLC-FLD post-column oxidation method [110,111] and a pre-column oxidation method [112,113]. These methods have been internationally validated and accepted and are now the AOAC 2005.06 and AOAC 2011.02 Official Methods of Analysis (OMA) [114,115]. Later studies extended the validation of AOAC 2005.06 OMA to dcGTX2,3 toxins and provided details on an hydrolysis procedure applicable to the quantification of GTX6, C3 and C4 [116,117]. In recent years, there have been significant advances in the use of LC with electrospray ionization mass spectrometry (ESI-MS) for the detection and quantification of PSTs in both phytoplankton and shellfish tissues [13]. A new hydrophilic interaction LC method with tandem mass spectrometry (MS) (HILIC-MS/MS) was developed for PSTs and tetrodotoxin analyses [118]. This method has also been internationally validated [16] and allows the detection and quantification of 19 PSTs congeners. 

##### Determination of PSTs in Seabird Samples by HPLC and LC-MS/MS Methods

To the best of our knowledge, one of the first reports of PSTs detected in bird samples by HPLC (method not specified) was a workshop presentation by Levasseur et al. [58] reporting an *A. tamarense* bloom in St. Lawrence estuary, Quebec (Canada). In the same area, mortalities of Sand Lance and Herring Gulls (*L. argentatus*) were described. The authors tested both dead Sand Lances and Herring Gulls (for levels, see Table 1). Gartrell et al. [119] employed the AOAC 2005.06 OMA [114] in order to investigate a mortality cluster in wild adult yellow-eyed penguins (*Megadyptes antipodes*) in New Zealand. The number of samples evaluated was very limited and they did not detect PSTs.

In the study conducted by Starr et al. [35], various carcass tissues were investigated for PSTs by ELISA, with selected samples tested by HPLC-FLD with post-column oxidation [110], with the aim of quantifying individual congeners. These results were confirmed by HPLC-MS/MS on an API 4000 Q-trap LC-MS (applied Biosystems) with Agilent 1200 HPLC using a triple quadrupole detector and ion-spray [120]. PST levels are reported in Table 1. 

In the [37] aforementioned study, seabird tissues (*n* = 3) and forage samples (*n* = 4) with levels > 70 µg STX equivalents·kg^−1^ by ELISA were tested by HPLC using the AOAC 2005.06 OMA [114]. The PSTs determined were: dcGTX2,3; C1,2; dcSTX; GTX2,3; GTX5; STX; GTX1,4; and NEO. Although STX was quantified in three invertebrate samples, none of the PSTs tested was detected in the seabird samples by HPLC. The authors suggest that these seabird tissues might have contained congeners with high HPLC detection limits. In another study, Van Hemert et al. (2021) [62] evaluated by the same HPLC method 6 samples that showed PSTs levels higher than 100 µg STX equivalents·kg^−1^ by ELISA. They quantified STX in all the samples, but other congeners were also present in 3 of them.

In a study to ascertain the cause of a paretic syndrome in gulls from southern Portugal [38], PSTs were tested in samples from ten gulls kidneys and in the cloaca contents from another gull by the AOAC 2011.02 OMA with slight modifications [121]. Although PSTs were not detected in the samples, it is important to highlight the presence of interferences in all of them. In order to confirm that these were naturally fluorescent compounds rather than PSTs an additional test consisting of re-running the samples without the oxidation step was implemented [22]. This step is highly recommended to avoid false positives when analyzing seabird samples by the AOAC 2011.02 OMA [115]. Further studies with the evaluation of additional samples by LC-MS/MS are ongoing in CEFAS, UK laboratory.

#### 6.1.3. Homogenization and Extraction Protocols: Adaption to Seabird Samples

The first step in the determination of PSTs is sample homogenization. Ideally, we should have a representative sample. In mollusks, this is achieved by selecting several specimens and obtaining a minimum sample size (generally 100 g tissue for mussels, oysters, clams, etc.) [105] or a dozen specimens for scallops. However, sampling size and homogenization of seabird tissues need adaption, due to the fact, that the target samples are organ tissues from death animals, that we generally analyze individual seabirds and that tissue sizes are very small [38]. In the determination of PSTs in seabird tissues, both by ELISA and HPLC, Van Hemert et al. tried to use 5 g homogenized samples as recommended in the extraction protocol [37]. However, they reported that it was not always possible to obtain this much material from seabird samples, and in those instances, dilution volumes and calculations were adjusted accordingly. The authors also point to the fact that very low STX concentrations may have been less consistently detectable in samples <5 g. In the studies conducted by Ben-Gigirey et al. [38], similar difficulties were experienced. Individual kidneys from each gull were supplied as duplicate samples. However, it was required to use both kidneys to obtain enough tissue, and for some gulls, it was not even possible to obtain 5 g of homogenate. Another difficulty experienced was the homogenization procedure itself. Although the smaller Ultraturrax^®^ dispersing tool was selected, the tissue stuck to the head was very difficult to collect and some sample was lost.

Extraction of PSTs from samples is usually conducted with an acidic solvent. In the AOAC 2005.06 OMA procedure, a duplicate extraction (first one with heating) with 1% acetic acid solution is employed. This is a mild extraction that maintains the original sample toxic profile, avoiding some PSTs hydrolysis into more toxic analogs. This procedure was used by Van Hemert et al. [37] for the testing of PSTs by ELISA and HPLC. In the MBA [105] and the AOAC 2011.02 OMA [115] protocols, homogenized samples are mixed with dilute 0.1 M hydrochloric acid and heated in a boiling water bath for 5 min. The mixture pH has to be carefully checked, and if necessary, adjusted between 2.0 and 4.0 (preferably 3.0), before and after the boiling step. The pH adjustment is critical, since lowering the pH below 3 could result in the hydrolysis of N-sulfocarbamoyl toxins into the more toxic carbamate analogs, with the subsequent increase in the sample total toxicity. This was the extraction protocol employed by Ben-Gigirey et al. (2021) [38] for the seagull kidney studies.

#### 6.1.4. Tissue Selection

There exists a lack of information about the metabolism and excretion of PSTs in seabirds. Some pharmacokinetic studies conducted in laboratory animals (rat and cats) are compiled in [24], which could be used as a guide. These studies suggest that STX is rapidly eliminated in urine, but the liver and gastrointestinal tract could be an alternate route of elimination and excretion in rats [24]. The fact that PST pharmacodynamics in birds are still unknown, makes it is difficult to assess ideal organs for the quantification of toxin levels [62]. The main tissues selected by different authors for the evaluation of PSTs in seabirds are liver [35,60,62,64], gastrointestinal (GI) tract parts (intestine, stomach, cloaca) or its contents [10,35,38,58,62,63,64,119] and kidney [35,38,60,64]. 

In a recent study [37], the authors followed a different approach depending on the bird conditions. In the case of birds found dead or lethally collected, they selected breast muscle, liver, upper GI contents (stomach contents and/or entire stomach and gizzard), and cloaca (entire cloaca and/or cloaca contents) for STX evaluation. For healthy, live captured birds, they took feces (kittiwakes and murres) and regurgitated samples (kittiwakes only). This non-invasive sampling procedure could be very useful to analyze PSTs in healthy or live animals with PSP suspected symptoms. In their study, the highest STX levels were found in the liver of die-off murres, whereas among healthy murres, STX was most prevalent in cloaca, but had the highest concentration in upper GI samples [37]. They also point to the fact that most die-off birds were emaciated and samples from the GI tract (where the toxin is typically most concentrated) were not available.

### 6.2. ASTs

DA, the main AST, is a water-soluble and heat-stable cyclic amino acid, neurotoxic and structurally very similar to kainic acid [122]. Several isomers of DA (epi-domoic acid (epi-DA), (domoic acid C5’-diastereomer) and isodomoic acids A, B, C, D, E, F, G and H (iso-DA A-H)) have been reported as well [123]. Iso-DA A, B and C have not been detected in shellfish tissue. DA transforms into epi-DA through long-term storage [124] and degrades and transforms to epi-DA and iso-DAs through exposure to ultraviolet light [125,126]. In addition, the epimerization is also accelerated by heating [127]. Biochemical and instrumental methods aim principally to detect DA, the main analogue.

#### 6.2.1. MBA

The first assays for DA detection were based on animal testing and were used to elucidate the first cases of DA poisoning in humans [128]. Mice injected with a sample containing DA exhibit characteristic symptoms, such as: scratching, loss of balance, sedation, rigidity, convulsions and death [129,130]. However, MBA for DA detection was early substituted for more sensitive instrumental and biochemical detection tools [124,131,132]. 

#### 6.2.2. ELISA for DA

Rapid biochemical assays to screen the presence of toxins in a collection of samples are also available. Among them, ELISA for DA detection is probably the most user friendly method, providing also very low LOD (3 µg·kg^−1^) [131,133,134]. 

While for complex matrices, such as seabird tissues, a clean-up procedure is recommended prior to the detection step, the elimination of the clean-up step may be possible for ELISA assays by sample dilution. However, the most appropriate dilution must be previously assessed to avoid matrix interferences without compromising the detection of the toxin [37,64,98,135,136,137]. This step was successfully used to detect DA in cloacal contents of common murres found stranded in California [98] and several tissues of white-winged scoters, double-crested and Brandt’s cormorants, ring-billed gulls, Pacific loons and Clark’s grebes involved in different outbreaks along the coasts of Washington, Rhode Island and California counties [64] (Table 2).

#### 6.2.3. Instrumental Methods for DA

The analytical method for DA detection based on high-performance liquid chromatography coupled with ultraviolet detection (HPLC-UV) is well established and widely used for marine biotoxins official controls [123,138], reaching LODs as low as 20 µg DA kg^−1^ [139,140]. An HPLC method with fluorescence detection after column derivatization (HPLC-FLD) was also developed with similar LOD [141,142]. The rapid advancements in analytical equipment have led to the improvement of sensitive tools based on LC-MS and LC-MS/MS. These methods, based on the identification of molecular weights and characteristic fragmentation patterns of the molecule, allow the detection of DA and its isomers with high specificity and sensitivity [14,36,143,144].

#### 6.2.4. Homogenization, Extraction and Clean-Up Protocols: Adaption to Seabird Samples

As previously stated for PSTs, the homogenization of avian tissues is complex and the amount of sample available is usually the main limitation. The same applies to DA analyses.

Extraction procedures for DA analyses may depend on the sample and detection technique employed. Most standardized protocols were designed to ensure seafood safety and they do not always match the requirements of complex matrices and fluids. For bivalve tissues, a 4 g pooled sample with 50% MeOH homogenization is employed, but this tissue amount may not be achieved when working with individualized seabird organs. A lack of specific methodologies for seabird tissues exists and a previous validation of extraction and detection techniques is necessary prior to the samples’ analyses.

One of the first methods for DA analysis in seabird samples involved an extraction step with HCl and LC with UV detection. It was employed prior to Quilliam’s method [139] and LC-MS for the analysis of stomach, digestive tract, cloaca contents and feces from sick and dead seabirds [47,48,145]. However, better DA recovery and stability are achieved with aqueous MeOH-based extraction methods [139]. Some samples could require an additional SPE clean-up step to avoid possible interferences (i.e., with strong anion exchange cartridges). If samples are analyzed by LC-UV, tryptophan could mimic the peak of DA. Therefore, the use of DA and tryptophan standards, together with monitorization at two different wavelengths, is recommended to check the proper separation between both compounds [139]. Confirmation of the toxin presence by other techniques like LC-MS or LC-MS/MS or performing a “blank” with healthy animal samples would be another way to discern between real and false positives in newly explored tissues. The extraction protocol developed by Quilliam et al. [139] with additional strong anion exchange (SAX) SPE clean-up was successfully applied for tissues and cloaca contents from seabirds [37,64,97,98].

An extraction protocol for urine and serum samples was developed using Oasis^®^ HLB extraction cartridge for HPLC-MS/MS detection [146]. The HLB polymeric sorbent provides very high recoveries with a simple protocol. This methodology was employed to measure DA in shearwaters’ whole blood, with an additional cleaning step using centrifuge filters [36]. By this method, detectable levels of DA ranging from 1–10.6 ng·mL^−1^ were identified in *Calonectris borealis* and *C. diomedea* (Table 1). The procedure may also be suitable for DA monitoring in seabird fecal samples and tissue extracts with slight modifications [14].

#### 6.2.5. Tissue Selection

Depuration rates have been shown to be relatively high for DA for a number of animal models, including birds, and therefore DA is usually found at higher levels in the kidney than in the liver, muscle or brain tissues [48,64,97,98,147]. Maximum DA levels were found in pigeons, ducks and common murre urates within 1–3 h after DA intracoleomic/oral administration [97]. DA was also detected in the kidney and was present at low levels in serum and livers [97].

For wild animals such as seabirds, the span of time between exposure to ASTs (and also PSTs) and death is usually unknown and ascertaining the cause of a mortality event is not always easy. In recently dead and sick animals, the analyses of stomach or digestive tract content and feces/urates may provide a first hint of the cause. The analyses of anchovies found in the stomach contents of sick and dead brown pelicans and Brandt’s cormorants revealed high quantities of DA in viscera and flesh [47]. Similarly, DA was found to be the cause of brown pelican mortalities in Mexico, after analysis of the digestive tract content, revealing *Pseudo-nitzschia* frustules and toxic chub mackerel [48]. A more recent episode involving common murres, in California, quantified DA in the stomach contents of several birds by LC-MS [98]. Similarly, a retrospective study performed in stranded birds in this area detected extremely high levels of DA in stomach contents of Brandt’s and double-crested cormorant from HAB events occurred in 2007 and 2015, respectively, as well as in Pacific loon, red-throated loons and Clark’s grebes collected after a *Pseudo-nitzschia* sp. bloom in 2017. The toxin was present also in white-winged scoter’s stomach content samples collected in 2009 [64]. On the other hand, DA causes vomiting, and poisoned animals may be unable to eat. Therefore, these samples are usually limited. The analysis of feces has been explored in seabirds to investigate the presence of other marine toxins such as brevetoxins [34], but has only been sporadically used for DA [37,97]. Cloaca contents from dead common murres revealed higher levels of DA than in kidney, stomach contents and liver from certain individuals [64,98]. This observation was confirmed in Pacific and red-throated loons and Clark’s grebes [98], pointing to excrements as a suitable sample to monitor DA prevalence in sick or healthy seabirds.

DA has been reported to be present entirely in the serum or plasma fraction, and thus, serum, plasma or whole blood could be taken for the analyses [147,148]. Recently, DA was detected in whole blood from GPS-tagged asymptomatic shearwaters in Spain [36]. The analyses of blood samples is an interesting approach since it would allow tracking DA in a non-invasive way in wild populations. Blood collection cards could have useful applications to store samples in the field, such as the simple protocol [148] designed to extract DA from mice blood stored in blood collection cards combined with a Biosense ELISA immunoassay.

## 7. Management and Prevention

MMEs are defined as 10 or more specimens of one species or species group found sick or dead, due to unknown causes, in the same place [149]. Birds tend to be very sensitive to marine abiotic and biotic (such as marine toxins) pollutants and have frequently provided the initial evidence for contaminants in local waters [29]. With regard specifically to HABs, since marine birds are predators that forage for prey offshore and then return to the coast, they are ideal sentinel species for monitoring the state of marine ecosystems [30]. Mortalities associated with HABs have been reported as an important cause of massive deaths in aquatic and marine species [17,30,32,64,100]. The increasing report of MMEs in seabirds over recent years [32,100,150,151] reinforces the importance of developing prevention and action plans. Prevention of huge mortality events is the most efficient and cheapest way to keep healthy wildlife populations [149], but environmental causes (like HABs) can be difficult to prevent. Large numbers of dead or dying seabirds can create an awareness of offshore marine events, and provide important clues of ecosystem disturbances. Seabirds are sentinel species in two ways [152]. First, they can serve as biomonitors of ecosystem scale changes, indicated, for instance, by the presence of abiotic pollutants in their tissues or marine litter (i.e., plastics) in their stomachs. Second, they can be quantitative indicators of ecosystem components such as fish, since their diet reflects the abundance of prey species within their foraging range [152]. For all these reasons, plans and protocols to improve the prevention, management and control of these episodes should be developed.

### 7.1. Entities Involved

The main entities that should get involved in the management and prevention plans should be governmental authorities, environmental non-governmental organizations (ENGOs), wildlife rescue hospitals, and the general public. An example of coordination among different coastal stakeholders is the Alaska Harmful Algal Bloom Network [153], a relatively new program that is attempting to bring together government agencies, ENGOs, and the public to address human and wildlife health risks from toxic algal blooms statewide. Their objectives include, among others, the expansion and enhancement of statewide HAB, wildlife and shellfish monitoring and the improvement of effectiveness of HAB events response.

#### 7.1.1. Governmental Authorities

Governmental authorities must develop national wildlife disease surveillance programs that include the prevention, action and investigation of cases. They should provide resources to other entities involved. 

In the U.S.A., we can find some examples of seabird’s MMEs management involving general public and wildlife rescue hospitals. The U.S.G.S. National Wildlife Health Center (USGS-NWHC) conducts diagnostic investigations to determine causes of wildlife (i.e., birds) morbidity and mortality events [154]. The Disease Investigation Services of the NWHC allow the submission of dead specimens from the general public, wildlife rescue hospitals, universities, private or zoo veterinarians, diagnostic laboratories or other entities [154]. Prior to responding to a mortality event, wildlife professionals should first consult with NWHC epidemiologists and, if applicable, their respective federal, state or tribal natural resources agency wildlife health program to discuss response options [154]. These wildlife disease experts can provide guidance on which specimens to collect and how to collect and best preserve specimens to maximize their diagnostic value. For this purpose, they have special protocols, such as Diagnostic Case Submission Guidelines, Wildlife Mortality Reporting and Diagnostic Services Request Worksheet and Instructions for Collecting and Shipment of Specimens all available at their website [154]. In our opinion, their program and protocols could be used as a guide. Useful information on beach surveys, specimens collection and preservation, how to record and submit specimens history data and necropsies protocols, can also be found in [10,33]. Since the NWHC capability to receive submissions from an MME is limited, they need collaboration to collect additional specimens or tissue samples for biotoxins analyses. This requires coordination with other entities, such as the U.S. Fish and Wildlife Service (USFWS). One of the USFWS responsibilities is to respond to incidents where a large number of birds are found sick or dead. USFWS has a complete Avian Mortality Event Response Plan for the Alaska region that reveals the importance of the cooperation between several agencies (Federal, Municipal, Tribal, State) and their different functions [149]. Another example of an avian mortality event evaluation in the U.S.A. is the Investigation of Persistent Seabird Mortalities along the Oregon Coast developed for the Environmental Contaminants Program of the USFWS [155]. The report by Materna et al. [155] analyzes the mortality of common murres between 1978 and 1997. Necropsies were performed and organic and inorganic residues were analyzed, concluding that, despite the presence of inorganic and organic compounds, the residues were not the cause of death. Starvation due to lack of food sources was identified as the cause of the MMEs [155]. 

The California Department of Fish and Wildlife runs a Seabird Health Program that provides a regional information center regarding marine bird mortality events for federal, state, and local resource managers [156]. The main program goals are to design and conduct studies to investigate and monitor the health and pathology of marine birds, to support the best achievable care of oiled wildlife and to detect emerging threats to seabird populations. They work collaboratively to gather regional data from beach survey programs, rehabilitation centers, and state and federal agencies. Information and contact details are available on their website [156].

In Europe, there exist standard protocols to act in suspected cases of avian flu and other zoonotic diseases, but the authors could not find any EU action protocol for governmental institutions in the case of bird MMEs. The measures to adopt on these situations rely on each country.

#### 7.1.2. Environmental Non-Governmental Organizations (ENGOs) 

ENGOs related to wildlife conservation or HAB events monitoring can provide information about species status, detection of mortality events or environmental threats as a result of their field studies. Furthermore, they can manage wildlife rescue hospitals or involve the general public in beach patrols. Some examples of ENGOs contributions are exposed in the general public and wildlife rescue hospitals sections. 

#### 7.1.3. Wildlife Rescue Hospitals

**Figure 2:**
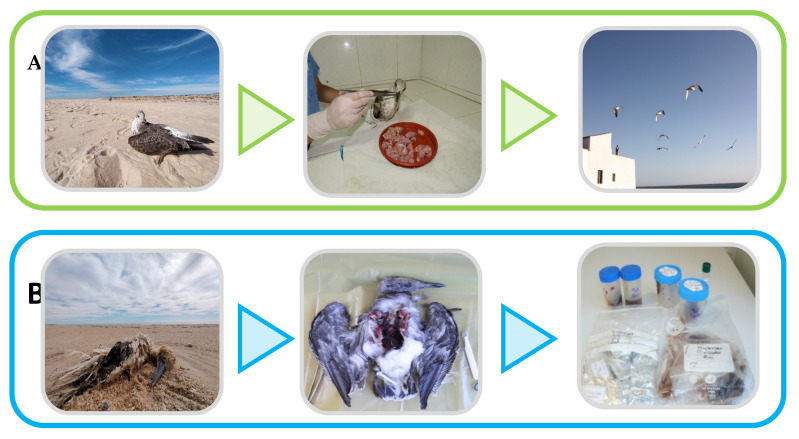
Schematic diagram of the actions taken at RIAS Wildlife Rehabilitation and Research Centre of Ria Formosa (Portugal) in case of finding beached seabirds and the processes involved afterwards. (**A**) alive birds are transported to the wildlife hospital where they are treated until fully recovered and released. (**B**) dead birds are taken to the wildlife hospital, necropsy center or laboratory where they are necropsied, and samples are taken in order to investigate the cause of death.

They can be the first line for the detection of seabird mortality and diseases. They should implement survey programs, the rehabilitation of affected birds and the investigation of the cases. A systematic and orderly data collection, in order to keep a long-term database with a complete pool of information, is desirable. The influence of existing local ENGOs or wildlife recovery centers may be crucial in the investigation and resolution of MMEs, but their financial difficulties are the major impediment to advance. As an example, we can mention the protocols followed at Wildlife Rehabilitation and Research Centre of Ria Formosa-RIAS, an animal hospital situated in the Ría Formosa Natural Park in the Algarve (Portugal). Staff from RIAS have been facing important and increasing gull admissions, associated with paretic syndrome, in recent years. Seabirds are delivered to RIAS by the general public who find them, or by Natural Park rangers. Animals that arrive alive are treated until they can be released back to nature. The gulls that die in the hospital or arrive dead are individually identified and frozen until necropsy is performed (Figure 2). The lack of financial support makes it very difficult to carry out specific analyses to confirm the cause of death. Staff from RIAS have been trying to establish collaborations with different research institutions, in order to send tissue samples for the analysis of botulism, marine biotoxins and cyanobacterial toxins, among others. As a result of this cooperation, marine biotoxins and botulism analyses were recently conducted in gull samples, in order to evaluate the causes of paretic syndrome [38].

**Figure 3:**
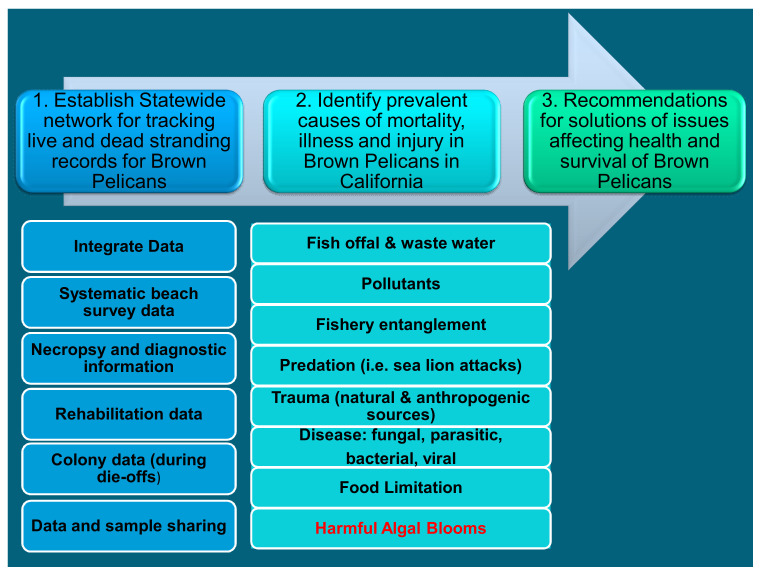
Main goals and interventions carried out by the Brown Pelican Mortality Working Group. Source: [157].

Another excellent example of how to tackle the issue of seabird mortalities is that of the Marine Wildlife Veterinary Care and Research Center (MWVCRC). The MWVCR, through the California Department of Fish and Wildlife’s Office of Oil Spill Prevention and Response and the University of California at Davis’ Oiled Wildlife Care Network, has provided state wide and regional post-mortem investigations for marine birds for over a decade [157]. The MWVCRC works in the assessment and sampling of marine birds during unusual mortality events and oil spills, in cooperation, among others, with wildlife rehabilitation centers, members of the National Wildlife Health Center, academic institutions and non-profit organizations. An example of their tasks was the creation of the Brown Pelican (BRPE) Mortality Working Group (WG) in 2013 that reviewed existing data to identify prevalent causes of mortality, illness and injury in BRPEs in California [157]. Other specific objectives of the WG were to establish a statewide network for tracking live and dead stranding records for BRPEs and to provide recommendations for solutions or the mitigation of issues affecting health and survival of BRPEs. Figure 3 summarizes the main goals and interventions carried out by the BRPE WG, as described in [157]. This example could guide other organizations in the development of future management plans. Their study concluded that the most significant mortality and morbidity factors for BRPEs in 2014 were fishery-related injuries and food limitation/malnutrition. However, historic necropsy data also revealed evidence of HAB intoxications due to *Pseudo-nitzschia* spp. producing DA, as reported in previous research studies [29,47,48]. Therefore, one of the WG main conclusions was that HABs should be monitored and the effects of marine biotoxins in BRPEs should be evaluated. 

#### 7.1.4. General Public

Frequently, the general public raises the first alarm about beached seabirds. Monitoring programs through citizen science or volunteering could help in these events’ detection [158,159,160]. A useful summary on how to act if finding birds with clinical signs or death that could be related to HABs can be found in [32]. This guidance (or its modifications to adapt to a particular area) could be provided to citizens.

The Center for Disease Control and Prevention (CDC) runs the One Health Harmful Algal Bloom System, a voluntary reporting system which collects data on human and animal illnesses caused by HABs together with environmental data about HABs [161]. This program, that involves the general public, allows collecting complete and ordered data, which are useful to study HAB impacts in wildlife over time. Their website includes a form and guidance to report individual or multiple cases of animal (including wildlife, such as birds) illness. The U.S.A. Coastal Observation and Seabird Survey Team (COASST) [162] is a Washington University citizen science project that focuses on the beach environment of the Northwest Pacific. COASST participants have contributed directly to monitoring their local marine resources and ecosystem health on more than 450 beaches from Northern California, Oregon, Washington, and Alaska. COASST is the largest beached bird network in the world and a very important resource for identifying and tracking die-off events. The Local Environmental Observer (LEO) Network is a global social media network that recruits citizen scientists to collect environmental observations on social media [163]. The Alaska Native Tribal Health Consortium Center for Climate and Health in Anchorage established the LEO Network web platform to allow tribal health workers and local observers to share information about environmental change. This is another valuable resource for collecting and collating information from on-the-ground observers. COASST and LEO are both excellent citizen science programs which are very effective in locations with remote coastlines where researchers cannot observe large portions of seabird habitats and therefore rely on local observers.

In Europe, the Iberian Group of Marine Birds (Grupo Ibérico de Aves Marinas-GIAM), formed by the Spanish Ornithology Society (SEO Birdlife) and the Portuguese Society for Avian Studies, developed an online application (Inspección de Aves Costeras Orilladas, ICAO), dedicated to involving the general public in a citizen science program to monitor the Iberian Peninsula coast for dead birds [164]. The program is still on an experimental phase and aims, among others, to identify the death causes in seabirds found on beaches and hence evaluate their preservation problems.

### 7.2. Prevention and Management Protocols

The Sea Alarm Foundation [165] is an example of integration between government, industry and ENGOs looking for the prevention and management of marine wildlife oiled emergencies and its methodology could be extrapolated to every seabird mass mortality or disease events. Established in 1999, Sea Alarm roots are in the European wildlife rehabilitation community. Since then they have organized and facilitated workshops worldwide, developed national oiled wildlife response plans in several countries, kept several phone lines on call 24 h, 7 days a week to receive notifications of oiled wildlife incidents and requests for assistance, provide distant advice and coaching, and to organize on-site visits and international resource mobilization when required [165]. Furthermore, they keep Country Profiles from more than 100 countries worldwide [166]. The structure of their Country Wildlife Response Profiles is based on the Country Profiles published by the International Tanker Owners Pollution Federation Limited (ITOPF) [167]. These profiles provide a summary of spill response arrangements and resources in maritime nations (Table 7). Similar profiles could be drawn with the organizations involved in seabird protection and experts in HABs and marine biotoxins fields to respond to MMEs.

The USFWS Avian Mortality Event Response Plan [149] involves preparation and response actions that could serve as guidelines in areas where such plans are unavailable. 

The preparation steps include:

1. Creating clear and easy ways to communicate the event. Communication channels are available to the general public and public agencies. There are several ways to communicate (phone numbers, email, online formulary, etc.).
2. Training personnel involved in wildlife health and disease response. The training is given both online and locally by the USFWS Wildlife Health Office to the Department of the interior employees, and wildlife hospitals workers, among others. It includes information on personal protective training and equipment.
3. Preparing first response kits that include, among others, personal protective equipment, important contacts list and material for collecting and packing the carcasses.

Once an event happens, the Avian Mortality Event Response Plan contemplates 4 response steps:

1. Reporting the event. They provide a list with all the contacts in each area.
2. Collecting basic information about the event: contact details of the person in the field, date of onset, exact location, etc. The response team records the exact number, species, sex and age of the carcasses, samples collected, preservation method and storage and symptoms shown in sick animals.
3. Collection, packaging and shipping the carcasses. Contacting the laboratory to assist in samples collection, packaging and storage. Collecting the freshest dead specimens that should be representative of all the affected species. Discarding carcasses appropriately to prevent scavenging.
4. Communicating the results in a direct an efficient way involving general public, national agencies, residents, wildlife hospitals staff, social media, etc.

The lack of human and logistic means is, in most cases, the main drawback to developing complete plans for seabirds MMEs in most countries. Unfortunately, regarding HAB seabird-related mortalities, biopsies, necropsies, and toxin analyses (in blood, tissues and feathers) are rarely conducted [32]. Therefore, cause/effect associations often rely on anecdotal evidence, which is insufficient for clearly implicating algal toxins as the causative agent [29,32]. From the previous sections, it can be inferred that the number of papers where PSTs and/or ASTs analyses have been conducted in seabird samples from MMEs is very limited. On top of that, sometimes analytical sample size is not representative for the total affected population [32]. Most of the studies consulted in this review mention a high number of death seabirds as opposed to a minimal number of samples analyzed. Another issue may be the methods selected for analysis. Early studies to analyze PSTs in seabirds were conducted with MBA, which is not sensitive enough to detect the very low PSTs levels that could cause seabird death [106]. Some of the more recent studies used ELISA kits that, although useful for screening purposes, do not cover most PSTs for which standards are available [107]. Furthermore, most seabird MMEs incidence reports are likely to severely underestimate the number of affected individuals [29] and this could be related to the sampling procedure employed (i.e., collecting only carcasses found in beaches). Sensitization of the general public in the potential harmful effects of HABs in seabirds and citizens involvement in the management of seabirds MMEs is important and should be part of a good management plan.

## 8. Conclusions

There exist difficulties in establishing the relationship between marine bird mortality events and HABs due to several factors such as lack of means and financial resources, specific and expensive laboratory tests, lack of structured and coordinated action protocols, absence of systematic history records and, in certain areas, very low interest in common species. In many remote areas, the lack of observers and difficult collection/sampling logistics could be an additional handicap. To better manage and ensure the future viability of seabird populations, it is imperative to investigate and incorporate the risks posed by HABs to seabirds in different world areas. This could include the proper design of the sampling plans, the implementation of standardized necropsy protocols in a representative number of dead seabirds, the collection of non-invasive samples from symptomatic and healthy birds, the adoption of protocols for the recovery of ill birds and the establishment of procedures for the systematic testing of potential algal biotoxins involved. 

In terms of PST and AST analysis in seabird samples, laboratories should preferably select sensitive methods that cover all the toxins for which standards are available. It is also required to adapt these methods (generally developed and validated for bivalve mollusks) to seabird matrixes and to their small sample sizes. Tissue selection is also a critical point. In terms of PSTs, the lack of studies on their metabolism and elimination on seabirds makes it more difficult to select the right tissue. The consideration of nonlethal sampling (feathers, blood or fecal analyses) should be further explored, since it could allow the analysis of marine biotoxins in more animals. The phytoplankton monitoring of the areas where MMEs are taking place is also crucial to aid in the selection of the potential marine biotoxins present in the samples.

It is important to highlight that the investigation of the cause/s behind MMEs requires a coordinated research effort at clinical, ecological and analytical levels. Support from governments and citizens collaboration are also essential. All together would allow a better understanding of the effects of HABs in wild bird populations and their implication on ecosystems health, permitting their use as sentinels in marine environments.

## Figures and Tables

**Table 1:** PSTs concentrations reported in seabird tissues (Conc.: concentration, <LOD= under the limit of detection, <LOQ=under the limit of quantification).

Species		Location, Year	Tissue	Conc. Ranges (μg STX·eq·kg^−1^)	Observations	Refs.
Scientific Name	Common Name					
*Alca torda*	Razorbill	St. Lawrence Estuary, Quebec, 2008	Digestive tract	<LOD–960	-	[35]
			Liver	<LOD–150	-	
*Ardea herodias*	Great blue heron	St. Lawrence Estuary, Quebec, 2008	Several tissues	<LOD	-	[35]
*Ardenna tenuirostris*	Short-tailed shearwater	Gambell and Shishmaref, North Berin Sea, Alaska, 2017	Several tissues	<LOD	-	[62]
		St. Paul Island, Pribilof Islands, Alaska, 2017	Stomach and cloaca contents	<LOQ	Pooled samples from several species	
			Liver	<LOD	-	
*Brachyramphus brevirostris*	Kittlitz’s murrelet	Kodiak Island, Alaska, 2011–2012	Upper gastrointestinal content	<LOD–216	Dead chicks. Values probably underestimated	[60]
			Liver	56.3–106.4		
			Kidney	27.9		
*Cepphus grylle*	Black guillemot	St. Lawrence Estuary, Quebec, 2008	Digestive tract	64–700	-	[35]
			Liver	<LOD–410	-	
*Fratercula cirrhata*	Tufted puffin	St. Paul Island, Alaska, 2016	Stomach and cloaca contents	3.1–9.5	Concentrations for each tissue not specified	[63]
*Fratercula corniculata*	Horned puffin	Shishmaref, North Berin Sea, Alaska, 2017	Stomach and cloaca contents	<LOQ	Pooled samples from several species	[62]
			Several tissues	<LOD	-	
*Fulmarus glacialis*	Northern fulmar	St. Lawrence Estuary, Quebec, 2008	Several tissues	<LOD	-	[35]
		Gambell and Shishmaref, North Berin Sea, Alaska, 2017	Cloaca and stomach contents	46.0	Pooled sample	[62]
			Stomach content	<LOQ–149	-	
			Stomach	12–53	-	
			Intestinal contents	21–111	-	
			Intestine	15–129	-	
			Liver	<LOQ–59	-	
			Muscle	<LOQ–15	-	
		St. Paul Island and St. George Island, Pribilof Islands, Alaska, 2017	Cloaca and stomach contents	46–305	Pooled sample	
			Stomach contents	<LOD–633	-	
			Intestine	<LOD–145	-	
			Liver	<LOD–44	-	
			Several tissues	<LOQ	-	
		San Luis Obispo County, California, 2018	Liver	6.9	-	[64]
			Kidney	8.8–9.6	-	
			Bile	21	-	
*Gavia immer*	Common loon	St. Lawrence Estuary, Quebec, 2008	Digestive tract	45, 19	Results from 1 sample. Conc. for ELISA and HPLC, respectively	[35]
			Liver	<LOD		
*Gavia stellate*	Red-throated loon	St. Lawrence Estuary, Quebec, 2008	Digestive tract	61	-	[35]
			Liver	<LOD	-	
*Hydrobates furcatus*	Fork-tailed Storm-petrel	Unalaska and Aleutian Islands, Alaska, 2017	Several tissues	<LOQ	-	[62]
*Larus argentatus*	Herring gull	St. Lawrence Estuary, Quebec, 1996	Intestine	110	-	[58]
			Brain	48	-	
		St. Lawrence Estuary, Quebec, 2008	Digestive tract	47–690	-	[35]
			Liver	100	-	
*Larus delawarensis*	Ring-billed gull	St. Lawrence Estuary, Quebec, 2008	Digestive tract	420	-	[35]
			Liver	<LOD	-	
		Providence County, Rhode island, 2016	Cloaca contents	<LOD	-	[64]
*Larus fuscus*	Black-backed gull	Ria Formosa, Olhão, Portugal, 2020	Several tissues	<LOD	-	[38]
*Larus marinus*	Great black-backed gull	St. Lawrence Estuary, Quebec, 2008	Several tissues	<LOD	-	[35]
*Larus michahellis*	Yellow-legged gull	Ria Formosa, Olhão, Portugal, 2020	Several tissues	<LOD	-	[38]
*Larus philadelphia*	Bonaparte’s gull	St. Lawrence Estuary, Quebec, 2008	Digestive tract	<LOD–31	Results from 1 sample. Conc. for ELISA and HPLC, respectively	[35]
*Larus sp.*	Gull (not identified)	St. Lawrence Estuary, Quebec, 2008	Liver	337	-	[35]
			Digestive tract	54.7	-	
*Melanita deglandi*	White-winged scoter	Grays Harbor County, Washington, 2009	Liver	<LOD–6.4	-	[64]
			Bile	<LOD–6.2	-	
			Several tissues	<LOD	-	
*Melanita perspicillata*	Surf scoter	Grays Harbor County, Washington, 2009	Intestinal contents	<LOD–4.7		[64]
*Morus bassanus*	Northern gannet	St. Lawrence Estuary, Quebec, 2008	Digestive tract	110–850	-	[35]
			Liver	850	-	
			Kidney	<LOD–63	-	
			Muscle	<LOD–87	-	
*Phalacrocorax auritus*	Double-crested cormorant	St. Lawrence Estuary, Quebec, 2008	Digestive tract	<LOD–370	-	[35]
			Liver	<LOD–58	-	
		Kent County, Rhode Island, 2016	Stomach contents	<LOD	-	[64]
*Phalacrocorax penicillatus*	Brandt’s cormorant	Marin County, California, 2015–2016	Stomach contents	<LOD–2.0	-	[64]
*Rissa tridactyla*	Black-legged kittiwake	St. Lawrence Estuary, Quebec, 2008	Digestive tract	<LOD–1340	-	[35]
			Digestive tract+liver	<LOD–520	-	
			Liver	<LOD–88	-	
		Gulf of Alaska, 2015–2017	Cloaca	<LOQ	-	[37]
			Upper gastrointestinal contents	46	-	
			Liver	27	Healthy animals. Minimum toxin level not provided	
			Muscle	37		
			Several tissues	<LOD	-	
*Somateria mollissima*	Common eider	St. Lawrence Estuary, Quebec, 2008	Digestive tract	<LOD–740	-	[35]
			Liver	<LOD	-	
*Sterna hirundo*	Common tern	Monomoy National Wildlife Refuge, Massachusets, 1978	Liver	<LOD	Fish vomited by birds accounted 970 μg STX equivalents·kg^−1^	[46]
*Uria aalge*	Common murre	St. Lawrence Estuary, Quebec, 2008	Several tissues	<LOD	-	[35]
		Clallam County, Washington, 2009	Stomach contents	<LOD	-	[64]
		Gulf of Alaska, 2015–2016	Proventriculus and cloaca	1.4–3.9	Toxin levels in each sample not specified	[10]
		Gulf of Alaska, 2015–2017	Cloaca	48	-	[37]
			Upper gastrointestinal contents	10	13 μg STX eq·kg^−1^ in healthy animals	
			Liver	108	Minimum toxin level not provided	
			Several tissues	<LOQ	-	
		Shishmaref and Unalakleet, North Berin Sea, Alaska, 2017	Cloaca and stomach content	<LOQ	Pooled samples from several species	[62]
			Several tissues	<LOD	-	
		Monterey County, California, 2018	Liver	<LOD	-	
			Kidney	<LOD–4.9	-	

**Table 2:** DA concentrations reported in seabird tissues (Conc.: concentration, <LOD = under the limit of detection, <LOQ = under the limit of quantification).

Species		Location	Tissue	Concentration Ranges (μg DA·kg^−1^)	Observations	Refs.
Scientific Name	Common Name					
*Aechmophorus clarkii*	Clark’s grebe	Monterey County, California, 2007	Cloaca contents	<LOD	-	[64]
		Santa Barbara County, California, 2017	Cloaca contents	111.2–681.2	-	
*Calonectris borealis*	Cory’s shearwater	Gran Canaria, Canary Island, Spain	Blood	1.1–10.1 *	Healthy animals	[36]
*Calonectris diomedea*	Scopoli’s shearwater	Menorca, Balearic Island, Spain	Blood	1–10.6 *	Healthy animals	[36]
*Fulmarus glacialis*	Northern fulmar	San Luis Obispo County, California, 2018	Liver	1.5	-	[64]
			Kidney	3.5–5.7	-	
			Bile	3.0	-	
*Gavia pacifica*	Pacific loon	Monterey County, California, 2007	Cloaca contents	<LOD–46100	-	[64]
		Ventura County, California, 2017	Kidney	<LOD–33446	-	
*Gavia stellata*	Red-throated loon	Monterey County, California, 2007	Cecal content	75,300	-	[64]
			Bile	<LOD	-	
		Ventura County, California, 2017	Liver	0.65–6850	-	
			Bile	82.5–49.7	-	
*Larus delawarensis*	Ring-billed gull	Providence County, Rhode island, 2016	Cloaca contents	4.5–5.3	-	[64]
*Larus fuscus*	Black-backed gull	Ria Formosa, Olhão, Portugal, 2020	Several tissues	<LOD	-	[38]
*Larus michahellis*	Yellow-legged gull	Ria Formosa, Olhão, Portugal, 2020	Several tissues	<LOD	-	[38]
*Melanita deglandi*	White-winged scoter	Grays Harbor County, Washington, 2009	Liver	<LOD–23.2	-	[64]
			Kidney	<LOD–16.5	-	
*Melanita perspicillata*	Surf scoter	Grays Harbor County, Washington, 2009	Intestinal contents	<LOD–11.1	-	[64]
*Pelecanus* *occidentalis*	Brown pelican	Santa Cruz County, California, 1991	Stomach contents	<LOD–27,900	-	[47]
		Cabo San Lucas, Baja California, 1996	Stomach contents	<LOD–142,850	.	[48]
			Digestive tract	37,170	.	
			Liver	<LOQ	-	
		Monterey County, California, 2007	Intestinal contents	14,600	-	[64]
			Several tissues	<LOD	-	
		Monterey County, California, 2015–2016	Cloaca contents	0.00–2847	-	
*Phalacrocorax auratus*	Double-crested cormorant	San Luis Obispo County, California, 2015–2016	Kidney	0.00–82.9	-	[64]
		Kent County, Rhode Island, 2016	Stomach contents	9.0	-	
*Phalacrocorax penicillatus*	Brandt’s cormorant	Santa Cruz County, California, 1991	Stomach contents	<LOD–48,000	-	[47]
		Monterey County, California, 2007	Cloaca contents	<LOD	-	[64]
			Stomach contents	4000–29,000	-	
		Marin County, California, 2015–2016	Stomach contents	2.36–1632	-	
		Los Angeles County, California, 2017	Stomach contents	6270–71150	-	
*Ptychoramphus aleuticus*	Cassin’s auklet	Humboldt County, California, 2017	Kidney	<LOD–86.4	-	[64]
*Rissa tridactyla*	Black-legged kittiwake	Gulf of Alaska, 2015–2017	Several tissues	<LOD	-	[37]
			Feces and regurgitants	<LOQ	Healthy animals	
*Uria aalge*	Common murre	Clallam County, Washington, 2009	Stomach contents	<LOD–12.1	-	[64]
		Santa Cruz County, California, 2015	Cloaca contents	<LOD–63.2	-	[32]
			Liver	<LOD–4.0	-	
			Stomach contents	5-36–10.8	-	
			Kidney	<LOD	-	
		San Luis Obispo County, California, 2015	Cloaca contents	5.0–654.1	-	
			Kidney	10.7	-	
			Liver	<LOD–915.8	-	
		Monterrey County, California, 2015	Cloaca contents	<LOD–64.1	-	
			Kidney	<LOD–31.5	-	
			Liver	<LOD–9.5	-	
		Marin County, California, 2015	Cloaca contents	<LOD–6.5	-	
		San Mateo County, California, 2015–2016	Liver	<LOD–915.8	-	
		Gulf of Alaska, 2015–2016	Proventriculus and cloaca	<LOD	-	[10]
		Gulf of Alaska, 2015–2017	Several tissues	<LOQ	-	[37]
			Feces	<LOD	-	
		Humboldt County, California, 2017	Liver	<LOD–97.9	-	
		Monterey County, California, 2018	Liver	0.00–4.9	-	
			Kidney	20.6–21.0	-	

* Units reported in ng. mL^−1.^

**Table 4:** Potential vectors and phytoplankton species involved in seabird mortality events associated with ASP outbreaks.

Vectors	Affected Birds, Place and Dates	Phytoplankton Species	Observations	References
Anchovies	Brown pelicans, Brandt’s cormorants; California, USA; September 1991	*Pseudo-nitzschia australis*	DA detected in seabirds and fish	[47,94]
Mackerel and sardines	Brown pelicans; Baja California, México; January 1996 and January 2004	*Pseudo-nitzschia* spp.	DA detected in seabirds and fish in 1996. Coincidence with sardine mortality and DA detected in dead dolphins in 2004	[48,68,95]
Mainly anchovies, (squids and mussels also possible)	Brandt’s cormorants, brown pelicans, pacific loons, red-throated loons; Monterey County, California, USA; March–May 2007	*Pseudo-nitzschia australis*	DA detected in seabirds	[64]
Mainly anchovies, (squids and mussels also possible)	Common murres, surf scoters, white-winged scoters; several Washington counties, USA; September–October 2009	*Pseudo-nitzschia* spp	DA detected in seabirds	[64]
Mainly anchovies, (squids and mussels also possible)	Brandt’s cormorants, brown pelicans, double-crested cormorants, common murres; several California counties, USA; July 2015–March 2016	*Pseudo-nitzschia* spp	DA detected in seabirds. In murres it could be a secondary death cause	[32,64,75]
Mainly anchovies, (squids and mussels also possible)	Double-crested cormorants, ring-billed gulls; Kent and Providence Counties, Rhode Island, USA; October 2016	*Pseudo-nitzschia* sp	DA detected in seabirds	[64]
Mainly anchovies, (squids and mussels also possible)	Brandt’s cormorants, Clark’s grebes, pacific loons, Red-throated loons, Cassin’s auklets, common murres; several California counties, USA; April–May and July–August 2017	*Pseudo-nitzschia* sp	DA detected in seabirds	[64]
Mainly anchovies, (squids and mussels also possible)	Common murres, northern fulmars; Monterey and San Luis Obispo Counties, California, USA; February 2018	*Pseudo-nitzschia* sp	DA detected in seabirds.	[64]

**Table 7:** Information contained in the ITOPF Country and Territory Profiles [167].

Headings	Containing Information
Spill Notification Point	National contact to communicate an event
Response Arrangements	One or more authorities responsible for coordination in case of an event. Different levels in the command chain depending on the event seriousness
Response Policy	National contingency plan establishing priorities and approved or forbidden measures
Equipment	Government and private equipment such as boats, skimmers, dispersants, etc., and who provides it
Previous Spill Experience	Oil natural disasters country history
Hazardous and Noxious Substances	Response arrangements for other marine disasters, not oil-related
Conventions	International environmental conventions joined by the country
Regional and Bilateral Agreements	Signed by the country
